# Two divergent immune receptors of the allopolyploid *Nicotiana benthamiana* reinforce the recognition of a fungal microbe-associated molecular pattern VdEIX3

**DOI:** 10.3389/fpls.2022.968562

**Published:** 2022-08-15

**Authors:** Nan Wang, Zhiyuan Yin, Yaning Zhao, Zhengpeng Li, Daolong Dou, Lihui Wei

**Affiliations:** ^1^Institute of Plant Protection, Jiangsu Academy of Agricultural Sciences, Nanjing, China; ^2^College of Plant Protection, China Agricultural University, Beijing, China; ^3^College of Plant Protection, Nanjing Agricultural University, Nanjing, China; ^4^Jiangsu Key Laboratory for Eco-Agricultural Biotechnology Around Hongze Lake, School of Life Sciences, Huaiyin Normal University, Huaian, China

**Keywords:** hybrid vigor, divergent evolution, LRR-RLP, ethylene-inducing xylanase (EIX), microbe-associated molecular pattern (MAMP)

## Abstract

The allotetraploid Solanaceae plant *Nicotiana benthamiana* contains two closely related receptor-like proteins (RLPs), NbEIX2 and NbRXEG1, which regulate the recognition of VdEIX3 and PsXEG1, respectively. VdEIX3, PsXEG1, and their homologs represent two types of microbe-associated molecular patterns (MAMPs) that are widespread in diverse pathogens. Here, we report that NbRXEG1 also participates in VdEIX3 recognition. Both *eix2* and *rxeg1* single mutants exhibited significantly impaired but not abolished ability to mediate VdEIX3-triggered immune responses, which are nearly abolished in *eix2 rxeg1* double mutants. Moreover, a dominant negative mutant of *eix2* that contains a 60 bp deletion failed to respond to VdEIX3 and could suppress VdEIX3-induced cell death in the wild-type *N. benthamiana*. Further phylogenetic analyses showed that NbEIX2 and NbRXEG1 are obtained from different diploid ancestors by hybridization. These results demonstrate that the allotetraploid *N. benthamiana* recognizes two types of MAMPs by two homologous but diverged RLPs, which provides a model in which an allopolyploid plant probably exhibits defense hybrid vigor by acquiring divergent immune receptors from different ancestors.

## Introduction

Diverse pathogens constantly attack the sessile plants, resulting in enormous yield losses worldwide. Breeding disease-resistant crops by hybridization is an important strategy for disease management (Nelson et al., 2018). Since polyploidy plants often exhibit better fitness and agriculturally important traits than their diploid parents, interspecific hybridization has been broadly used in crop breeding to take advantage of hybrid vigor (Goulet et al., 2017), such as wheat, cotton, soybean, and oilseed rape. Studies have demonstrated that polyploidy confers increased resistance to pathogens. For instance, synthetic polyploidy garden impatiens resulted in significantly increased resistance to downy mildew (Wang et al., 2018a). However, the molecular basis of defense hybrid vigor in polyploidy plants remains largely elusive.

To withstand attacks by pathogens, plants have evolved a two-tiered immune system that detects pathogens and activates immune responses (Ngou et al., 2022). The cell-surface localized pattern recognition receptors (PRRs) and intracellular nucleotide-binding leucine-rich repeat receptors (NLRs) are immune receptors responsible for pathogens recognition. PRR- and NLR-coding genes are widely used in crop breeding to improve disease resistance. However, hybridization often leads to detrimental phenotypes, such as hybrid necrosis caused by autoimmunity that incurs growth penalties (Calvo-Baltanás et al., 2021). Recent studies have revealed that hybrid incompatibility is mainly caused by the inappropriate activation of immune receptors (Li and Weigel, 2021). Polyploidy plants usually have more immune receptors than their diploid progenitors, which is a consequence of whole-genome duplication (King et al., 2012). Duplicated immune receptors that confer hybrid vigor for disease resistance are rarely reported.

*Nicotiana benthamiana* is an important model plant used for molecular research and biotechnology worldwide. This Solanaceae plant is a native Australian allotetraploid species, whose progenitors arrived approximately 20 Mya (Goodspeed, 1947). Subsequent harsh climatic conditions probably drove the hybridization of progenitors, which produced *N. benthamiana* that survives in the drought environment (Bally et al., 2018). Recently, two closely related receptor-like proteins (RLPs) from *N. benthamiana*, NbEIX2 and NbRXEG1, were shown to recognize fungal xylanase and XEG1, respectively (Wang et al., 2018b; Yin et al., 2021), which are two types of MAMPs (microbe-associated molecular patterns). The tomato (*Solanum lycopersicum*) has two homologous RLPs, SlEIX1 and SlEIX2 (Ron and Avni, 2004). Among them, only SlEIX2 is required for fungal xylanase-triggered immunity (Ron and Avni, 2004), while SlEIX1 is a decoy receptor that attenuates SlEIX2 signaling (Bar et al., 2010). Our previous phylogenetic analysis revealed that NbEIX2 and SlEIX2 are orthologs, while NbRXEG1 is closely related to SlEIX1 (Yin et al., 2021). The distinct functions between NbRXEG1 and SlEIX1 suggest the potential divergent evolution of this RLP family in different Solanaceae plants.

Leucine-rich repeat (LRR) RLPs are a distinctive type of PRR that homologous RLPs only occur within a few related species (Guo et al., 2011). Likewise, NbEIX2 and its homologs are only present in a few Solanaceae plants: tomatoes, *Nicotiana* spp., and *Capsicum annuum* (Yin et al., 2021). Among these plants, diploid *S. lycopersicum*, *C. annuum*, and *N. tomentosiformis* have two homologs of NbEIX2 (Yin et al., 2021). The diploid *N. sylvestris* and allotetraploid *N. tabacum* have one and three homologs, respectively (Yin et al., 2021). *N. tabacum* is a hybrid of *N. sylvestris* and *N. tomentosiformis* (Leitch et al., 2008), which is consistent with the numbers of NbEIX2 homologs in these three species (1 + 2 = 3). The phylome analysis indicated that members of *Nicotiana* sections *Sylvestres* and *Noctiflora* are the paternal and maternal progenitors of *N. benthamiana*, respectively (Schiavinato et al., 2020). However, the current version of *N. benthamiana* genome only has two homologs, NbEIX2 and NbRXEG1, although *N. benthamiana* is an allotetraploid.

In this study, using single and double mutants of *eix2* and *rxeg1* generated by CRISPR/Cas9, NbRXEG1 was found to be also required for the recognition of fungal xylanase. Phylogenetic analyses demonstrated that the diverged NbEIX2 and NbRXEG1 were obtained from different ancestors. These findings suggest that a family of RLPs from the allotetraploid *N. benthamiana* confers redundant innate immunity, and provide a model in which a polyploidy plant exhibits defense hybrid vigor by acquiring divergent immune receptors from different ancestors.

## Materials and methods

### Plant materials and growth

*Nicotiana benthamiana* mutants *eix2-1*, *eix2-2*, *eix2-3*, *rxeg1-1*, *rxeg1-2*, *er-1* (*eix2 rxeg1*), and *er-2* were generated by CRISPR/Cas9. All plants were grown under long-day conditions (16 h light) at 25°C. Leaves from 5-week-old plants were used for all the assays in this study, including pathogen inoculation, transient protein expression by agroinfiltration, and determination of immune responses induced by VdEIX3.

### Bioinformatics analyses

NbEIX2 homologs from other Solanaceae plants were identified from the scaffold assemblies by BLAST. These protein sequences were aligned using CLUSTALW.^1^ The poorly aligned regions were removed by trimAl (Capella-Gutiérrez et al., 2009). The maximum likelihood phylogenetic tree was generated using the online phylogenetic tool W-IQ-TREE (Trifinopoulos et al., 2016), and the tree was visualized by iTOL (Letunic and Bork, 2021).

### Genome editing in *Nicotiana benthamiana* by CRISPR/Cas9

For genome editing by CRISPR/Cas9, guide RNAs targeting the coding sequence were designed using the online tool CCTop (Stemmer et al., 2015). Gene-specific guide RNAs were cloned into the pHEE401 vector according to the previous protocol (Wang et al., 2015). *Agrobacterium*-mediated transformation in *N. benthamiana* was carried out as described previously (Ellis et al., 1987). The edited genes of T0 plants were determined by PCR and sequencing. The homozygous T2 plants were used for subsequent studies. Primers used for genome editing are listed in Supplementary Table 1.

### Agroinfiltration in *Nicotiana benthamiana*

Genes used for transient expression in *N. benthamiana* leaves were cloned into the pCAMBIA-1300 vectors with different tags. Plasmid constructions were transformed into *Agrobacterium tumefaciens* GV3101. *A. tumefaciens* then cultured at 28°C for 16 h, and adjusted to a final OD_600_ of 0.6 using the infiltration buffer (10 mM MgCl_2_, 10 mM MES, 200 μM acetosyringone, pH 5.7).

*Agrobacterium tumefaciens* cells were infiltrated into *N. benthamiana* leaves using a syringe without needle. Primers used for transient expression are listed in Supplementary Table 1.

### Co-immunoprecipitation assays

Proteins expressed in *N. benthamiana* leaves were extracted using the lysis buffer (150 mM NaCl, 1 mM EDTA, 25 mM Tris–HCl pH = 7.5, 10% Glycerol, 2% PVP40, 1× protease inhibitor, 0.1% Triton X-100). After centrifugation at 15,000 rpm, 4°C for 15 min, the supernatant was transferred to a new tube containing 15 μL anti-FLAG beads (Sigma-Aldrich, A2220), and was slowly shaken at 4°C for 2 h. The beads were then washed three times using TBST and proteins were eluted by heating at 100°C for 10 min. The immunoprecipitated products were detected by immunoblot with anti-HA or anti-FLAG antibodies.

### Relative expression of pattern-triggered immunity marker genes

Recombinant proteins expressed in *Escherichia coli* were obtained as described previously (Yin et al., 2021). The 1 μM recombinant protein was infiltrated into *N. benthamiana* leaves, and total RNA was extracted using the Plant Total RNA Kit (ZomanBio, Beijing). Relative expression of PTI marker genes of *N. benthamiana*, *NbCYP71D20*, and *NbPTI5*, was determined by real-time qPCR as described previously (Yin et al., 2021). *NbACT* was used as the internal reference. Primers used for qPCR are listed in Supplementary Table 1.

### Pathogen infection assay

*Phytophthora capsici* LT263 was grown on the V8 medium in the dark at 25°C. Mycelial plugs with 5 mm diameters were collected by a cork-borer and inoculated on detached *N. benthamiana* leaves that were pre-treated with 1 μM recombinant protein for PBS buffer for 24 h. The inoculated leaves were placed in plastic boxes with high humanity in the dark. Photos of lesions caused by *P. capsici* were taken 36 h post-inoculation and the areas were measured by ImageJ. Finally, the genomic DNA of infected tissues was extracted and subjected to calculate relative *P. capsici* biomass using qPCR. Primers used for biomass analysis are listed in Supplementary Table 1.

### Accession numbers

The sequences of NbEIX2 homologs were retrieved from the GenBank database. Accession numbers are shown in the Figures.

## Results

### Fungal xylanases-induced immune responses are impaired but not abolished in the *eix2* mutants

**FIGURE 1:**
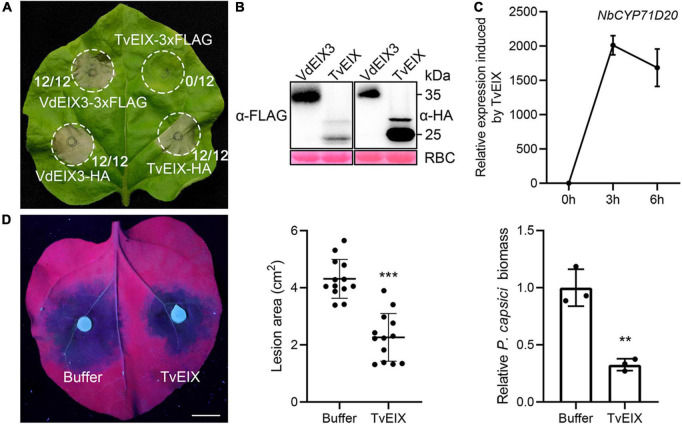
*Trichoderma viride* ethylene-inducing xylanase (TvEIX) induces cell death and immune responses in *Nicotiana benthamiana*. **(A)** The carboxyl-terminal epitope fusions influence the cell death-inducing activity of TvEIX in *N. benthamiana*. The fraction numbers indicate the number of infiltrated leaves that have cell death phenotype/total number of infiltrated leaves. **(B)** Proteins that were transiently expressed in *N. benthamiana* leaves were detected by immunoblot using anti-HA antibody. Ponceau S staining of Rubisco (RBC) indicates the loading control. **(C)** TvEIX protein activates the expression of PTI marker gene *NbCYP71D20*. **(D)** Pre-treatment of *N. benthamiana* leaves by 500 nM TvEIX protein significantly enhances plant resistance to the oomycete pathogen *Phytophthora capsici*. Photos were taken 36 h post inoculation under UV light. Data are shown as mean ± SD (Student’s *t*-test, ***P < 0.001). Bar, 1 cm. Relative *P. capsici* biomass in infected leaves was determined by qPCR (Student’s *t*-test, **P < 0.01).

About three decades ago, the *Trichoderma viride* ethylene-inducing xylanase (TvEIX) was identified as an elicitor that induces immune responses in common tobacco (*N. tabacum*) (Fuchs et al., 1989). EIX-like proteins are widely distributed in bacteria and fungi, several of which were also found to exhibit elicitor activity (Yin et al., 2021). However, what is puzzling is that TvEIX triggers cell death in tomato and *N. tabacum* but not in *N. benthamiana* (Ron and Avni, 2004), although *N. benthamiana* has a closely related RLP that can recognize VdEIX3, an EIX-like protein from *Verticillium dahliae* (Yin et al., 2021). Unexpectedly, we found that carboxyl-terminal epitope fusions influence the elicitor activity of TvEIX in *N. benthamiana*. When 3xFLAG-tagged TvEIX was transiently expressed in *N. benthamiana* by agroinfiltration, TvEIX failed to induce cell death as described by Ron and Avni (2004). The cell death-induced by TvEIX-1xHA is comparable to the positive control VdEIX3 (Figure 1A). Immunoblot analyses confirmed that both TvEIX-3xFLAG and TvEIX-1xHA were expressed normally in *N. benthamiana* (Figure 1B). To further determine its elicitor activity, the TvEIX recombinant protein expressed in *Escherichia coli* was infiltrated into the *N. benthamiana* leaves. The transcript level of *NbCYP71D20*, a marker gene of pattern-triggered immunity (PTI), was greatly elevated 3 h after TvEIX treatment (Figure 1C). Furthermore, the oomycete pathogen *Phytophthora capsici* caused significantly smaller lesions in *N. benthamiana* leaf regions pretreated by the TvEIX protein than that by buffer control (Figure 1D). Consistently, the relative *P. capsici* biomass in TvEIX-treated regions was significantly lower than that in buffer-treated regions (Figure 1D). These results indicate that TvEIX activates defense responses in *N. benthamiana*.

**FIGURE 2:**
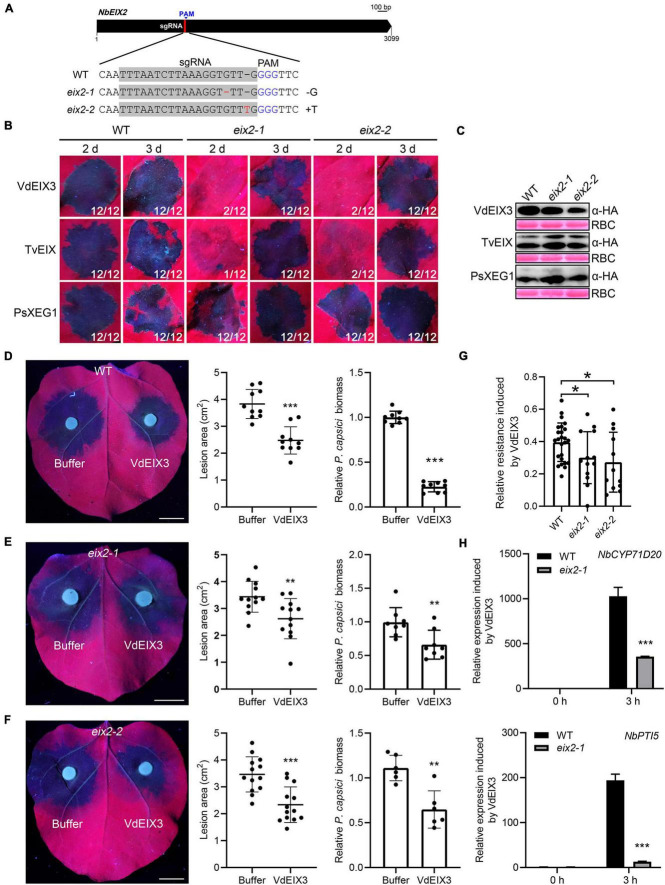
VdEIX3-induced immune responses are impaired but not abolished in the *eix2* mutants. **(A)** Sequence alignment of the region in *eix2* targeted by sgRNA. The protospacer adjacent motif (PAM) is marked in blue, and the edited nucleotides are marked in red. **(B)** VdEIX3 and TvEIX induce delayed cell death in *eix2* mutants. The photos were taken under UV light. **(C)** Proteins that were transiently expressed in *Nicotiana benthamiana* leaves were detected by immunoblot using anti-HA antibody. Ponceau S staining of Rubisco (RBC) indicates the loading control. **(D–F)** Pre-treatment of *N. benthamiana* leaves by 500 nM VdEIX3 for 24 h significantly promoted resistance to *Phytophthora capsici* in wild-type and *eix2* mutants. Photos were taken 36 h post inoculation under UV light. Data are shown as mean ± SD (Student’s *t*-test, ***P < 0.001, **P < 0.01). Bars, 1 cm. Relative *P. capsici* biomass in infected leaves was determined by qPCR (Student’s *t*-test, ***P < 0.001, **P < 0.01). **(G)** Relative resistance-induced by VdEIX3 protein in wild-type *N. benthamiana* and *eix2* mutants. Relative resistance is calculated by dividing **(A,B)** into A. **(A,B)** indicate lesion area of buffer-treated region and VdEIX3-treated region, respectively. Data are shown as mean ± SD (Student’s *t*-test, *P < 0.05). **(H)** Relative expressions of PTI (pattern-triggered immunity) marker genes induced by VdEIX3 protein in wild-type and *eix2* mutants. Data are shown as mean ± SD (Student’s *t*-test, ***P < 0.001).

NbEIX2 and SlEIX2 are two closely related RLPs that recognize VdEIX3 and TvEIX, respectively (Ron and Avni, 2004; Yin et al., 2021). Since TvEIX could also be recognized by *N. benthamiana*, to investigate whether NbEIX2 regulates the perception of TvEIX, we generated *eix2* mutants by CRISPR/Cas9. Two sgRNAs were designed to target the coding sequence of NbEIX2, only one of which led to successful genome editing (Figure 2). We obtained two homozygous *eix2* lines, which contain a 1 bp deletion (*eix2-1*) and insertion (*eix2-2*) that result in premature termination, respectively (Figure 2A and Supplementary Figure 1). Then, TvEIX and VdEIX3 were expressed in *eix2* mutants by agroinfiltration, while PsXEG1 which is recognized by NbRXEG1 but not by NbEIX2 was used as a control (Wang et al., 2018b). Consistently, PsXEG1 induced cell death in the wild-type *N. benthamiana* and *eix2* mutants (Figure 2B). Both TvEIX and VdEIX3 failed to induce cell death 2 days post infiltration in *eix2* mutants, while the wild-type *N. benthamiana* showed an apparent cell death phenotype (Figure 2B). Immunoblot analyses confirmed that all proteins were expressed normally in *N. benthamiana* leaves (Figure 2C). Unexpectedly, both TvEIX and VdEIX3 eventually induce cell death in *eix2* mutants 3 days post infiltration (Figure 2B). This finding indicates that xylanases-induced cell death was delayed but not abolished in the *eix2* mutants, which conflicts with our previous report that virus-induced gene silencing of *NbEIX2* abolished VdEIX3-triggered cell death in *N. benthamiana* (Yin et al., 2021).

To further test whether other immune responses induced by VdEIX3 are impaired or abolished in the *eix2* mutants, we firstly examined VdEIX3-induced resistance to *P. capsici*. In line with our previous study (Yin et al., 2021), pre-treatment of *N. benthamiana* leaves by VdEIX3 protein resulted in significantly smaller lesions and lower biomass caused by *P. capsici* (Figure 2D). When *eix2* mutants were pre-treated by VdEIX3 protein, *P. capsici* also caused significantly smaller lesions and lower biomass in VdEIX3-treated regions than that in buffer control (Figures 2E,F). To compare the relative resistance induced by VdEIX3 between wild-type *N. benthamiana* and *eix2* mutants, we calculated the inhibition rate by dividing the lesion area in the VdEIX3-treated region by that in control. The relative resistance induced by VdEIX3 in *eix2* mutants is significantly lower than that in wild-type *N. benthamiana* (Figure 2G), indicating that VdEIX3-triggered resistance to the pathogen is also impaired but not abolished in the *eix2* mutants. To further confirm that NbEIX2 partially contributes to xylanase recognition, the transcript levels of PTI marker genes *NbCYP71D20* and *NbPTI5* were also determined. Both *NbCYP71D20* and *NbPTI5* were remarkably activated in the wild-type *N. benthamiana* 3 h after VdEIX3 treatment. Likewise, their transcript levels in *eix2* mutants were significantly lower (Figure 2H). Collectively, these data indicate that fungal xylanases-induced immune responses are impaired but not abolished in the *eix2* mutant.

### The *eix2-3* mutant blocks VdEIX3-triggered immunity

**FIGURE 3:**
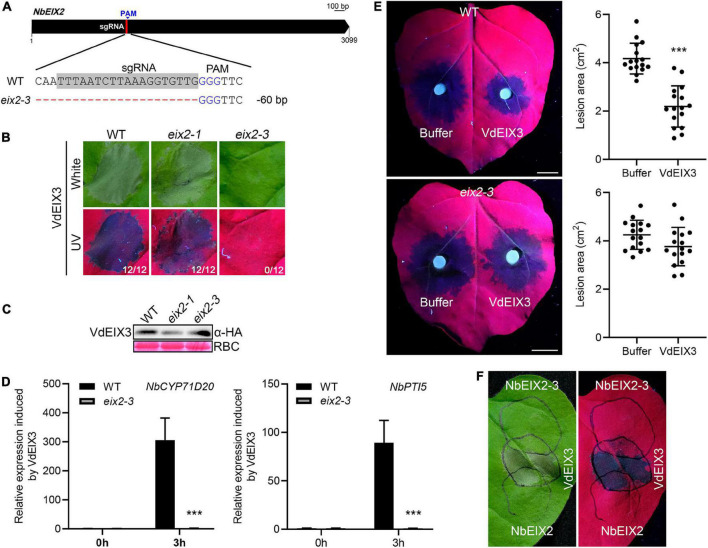
The dominant negative mutant *eix2-3* blocks VdEIX3-triggered immunity. **(A)** The *eix2-3* mutant contains a 60 bp deletion. The sgRNA region is highlighted in gray. **(B)** VdEIX3 fails to induce cell death in the *eix2-3* mutant by agroinfiltration. The photos were taken 3 days post infiltration under white and UV light, respectively. **(C)** Proteins expressed in *Nicotiana benthamiana* leaves were detected by immunoblot using anti-HA antibody. **(D)** Relative expressions of *NbCYP71D20* and *NbPTI5* induced by VdEIX3 protein in wild-type and *eix2-3* mutant. Data are shown as mean ± SD (Student’s *t*-test, ***P < 0.001). **(E)** VdEIX3 protein fails to promote resistance to *Phytophthora capsici* in the *eix2-3* mutant. Bars, 1 cm. Data are shown as mean ± SD (Student’s *t*-test, ***P < 0.001). **(F)** Transient expression of NbEIX2-3 in the wild-type *N. benthamiana* suppresses VdEIX3-induced cell death. NbEIX2 and NbEIX2-3 were expressed 24 h before VdEIX3. The photograph was taken 3 days post infiltration under white and UV light, respectively. Black circles indicate infiltration regions.

In the effort to generate *eix2* mutants, we obtained a third mutant *eix2-3* that contains a 60 bp deletion, which results in a truncated NbEIX2 that lacks 20 amino acids (Figure 3A and Supplementary Figure 1 and Supplementary File 1). Interestingly, when transiently expressed in the *eix2-3* mutant, VdEIX3 failed to induce cell death (Figure 3B). The protein was expressed normally in the *eix2-3* mutant as shown by the immunoblot analysis (Figure 3C). Furthermore, *NbCYP71D20* and *NbPTI5* activation induced by VdEIX3 protein were abolished in the *eix2-3* mutant (Figure 3D). We next determined VdEIX3-induced resistance to *P. capsici* in the *eix2-3* mutant. The result showed that pre-treatment of *eix2-3* leaves by the VdEIX3 protein resulted in comparable lesions to the buffer control (Figure 3E). These findings led us to hypothesize that NbEIX2-3 likely functions as a dominant negative mutant. As expected, transient expression of NbEIX2-3 in the wild-type *N. benthamiana* could suppress VdEIX3-triggered cell death, while NbEIX2 did not influence (Figure 3F). Taken together, NbEIX2-3 is likely a dominant negative mutant that blocks VdEIX3-triggered immunity, probably by competing with another unknown receptor that is also involved in VdEIX3 perception.

### NbRXEG1 is also involved in VdEIX3 recognition by *Nicotiana benthamiana*

**FIGURE 4:**
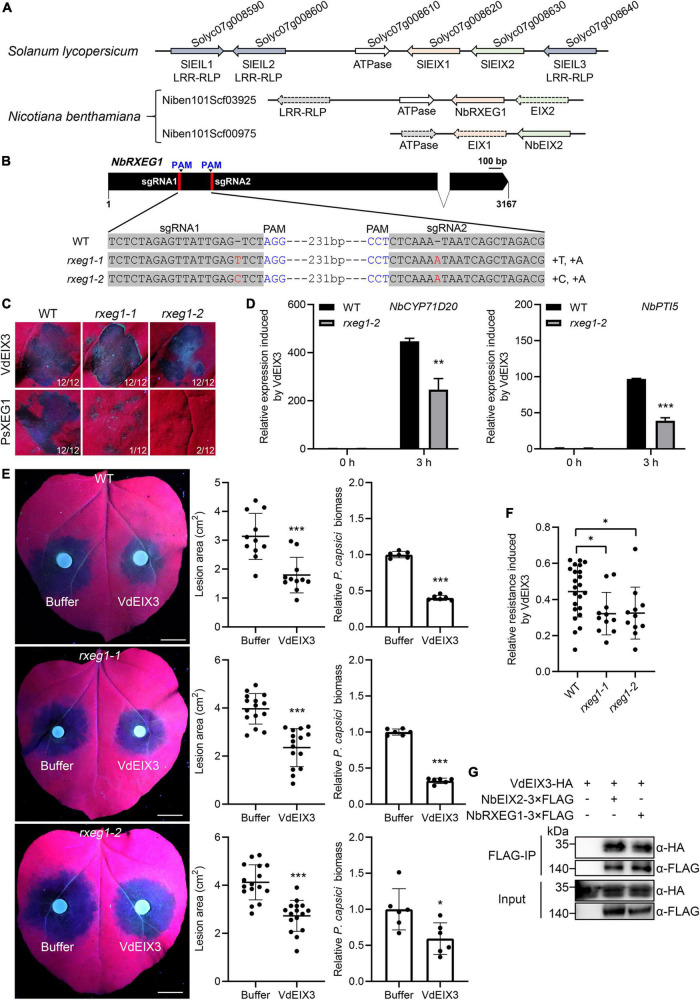
NbRXEG1 is also involved in the VdEIX3 recognition by *Nicotiana benthamiana*. **(A)** NbEIX2 homologs in the EIX-responding locus of tomato and *N. benthamiana*. The dotted arrow indicates pseudogene. **(B)** Sequence alignment of the regions in *rxeg1* targeted by sgRNAs. **(C)** VdEIX3 induces cell death in *rxeg1* mutants by agroinfiltration. The photos were taken 3 days post infiltration under white and UV light, respectively. **(D)** The transcript levels of *NbCYP71D20* and *NbPTI5* were reduced by half in the *rxeg1-1* mutant. Data are shown as mean ± SD (Student’s *t*-test, **P < 0.01). **(E)** VdEIX3-induced resistance to *Phytophthora capsici* was impaired in *rxeg1* mutants. Bars, 1 cm. Data are shown as mean ± SD (Student’s *t*-test, ***P < 0.001). **(F)** Relative resistance-induced by VdEIX3 protein was lower in *eix2* mutants than that in wild-type *N. benthamiana*. Data are shown as mean ± SD (Student’s *t*-test, *P < 0.05). **(G)** NbRXEG1 associates with VdEIX3 in co-immunoprecipitation (Co-IP) assay. VdEIX3-HA was co-expressed with NbRXEG1-3xFLAG in *N. benthamiana*. The immunoprecipitated product obtained by anti-FLAG beads was analyzed by immunoblot with anti-FLAG or anti-HA antibody.

Leucine-rich repeat-RLPs often display significant clustering in the plant genome (Guo et al., 2011), as is the case for the EIX-responding locus of *S. lycopersicum*, which contains two tandem LRR-RLPs, SlEIX1 and SlEIX2 (Ron and Avni, 2004). By searching their adjacent proteins, we found another three EIX-like (EILs) LRR-RLPs that share high similarity with SlEIX1 and SlEIX2 (Figure 4A). Therefore, we tried to identify additional PRR(s) required for VdEIX3 recognition by analyzing the NbEIX2 adjacent proteins. We found two scaffolds that contain NbEIX2 homologs, Niben101Scf03925 and Niben101Scf00975 (Figure 4A). Niben101Scf03925 contains NbRXEG1 and two NbEIX2 homologs that bear premature termination nevertheless. Likewise, Niben101Scf00975 contains NbEIX2 and a NbRXEG1-like pseudogene. This finding suggests that only NbEIX2 and NbRXEG1 are completed proteins in the EIX-responding locus of *N. benthamiana*, which prompted us to test whether NbRXEG1 participates in VdEIX3 recognition.

We thus generated *nbrxeg1* mutants by CRISPR/Cas9 and obtained two homozygous lines, which contain a 1 bp insertion in both sgRNA regions (Figure 4B and Supplementary Figure 1). Both *rxeg1-1* and *rxeg1-2* led to premature termination of NbRXEG1. We next investigated VdEIX3-triggered immunity in *rxeg1* mutants. Transient expression of PsXEG1 failed to induce cell death in *rxeg1* mutants 3 days post infiltration, indicating that *rxeg1* mutants lose the function of PsXEG1 detection (Figure 4C). However, the wild-type *N. benthamiana* and *rxeg1* mutants exhibited comparable cell death phenotypes induced by VdEIX3 (Figure 4C).

To our surprise, VdEIX3 protein treatment led to about 400-fold up-regulation of *NbCYP71D20* and *NbPTI5* in the wild-type *N. benthamiana*, while the relative expressions were reduced nearly by half in the *rxeg1-1* mutant (Figure 4D). Furthermore, when pre-treating *rxeg1* mutants with VdEIX3 protein, the average lesion areas caused by *P. capsici* were larger than that in the wild-type *N. benthamiana* although VdEIX3 significantly promoted plant resistance to *P. capsici* (Figure 4E). In line with the *eix2* mutants, *rxeg1* mutants showed significantly lower relative resistance by VdEIX3 treatment, compared to the wild-type *N. benthamiana* (Figure 4F).

The genetic evidence above confirmed that NbRXEG1 is also required for VdEIX3 detection. We further determined whether NbRXEG1 can associate with VdEIX3 by co-immunoprecipitation assay. In the immunoprecipitated product of *N. benthamiana* leaves co-expressing NbRXEG1-3xFLAG and VdEIX3-HA, VdEIX3 signal was detected by immunoblot with anti-HA antibody (Figure 4G), indicating that NbRXEG1 associates with VdEIX3 *in vivo*. Taken together, these findings above demonstrate that NbRXEG1 is also involved in the VdEIX3 recognition by *N. benthamiana*.

### The *eix2 rxeg1* double mutants are greatly impaired in regulating VdEIX3-triggered immunity

**FIGURE 5:**
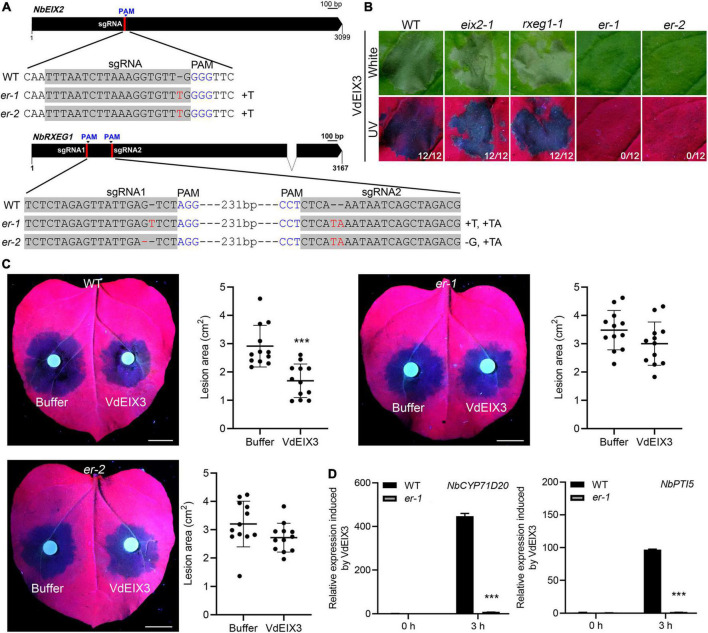
The *eix2 rxeg1* double mutants are greatly impaired in regulating VdEIX3-triggered immunity. **(A)** Sequence alignment of the regions in *eix2* and *rxeg1* targeted by sgRNAs. *er* indicates *eix2 rxeg1* double mutant. **(B)** VdEIX3 fails to induce cell death in *er* mutants by agroinfiltration. The photos were taken 3 days post infiltration under white and UV light, respectively. **(C)** VdEIX3 protein fails to promote resistance to *Phytophthora capsici* in *er* mutants. Bars, 1 cm. Data are shown as mean ± SD (Student’s *t*-test, ***P < 0.001). **(D)** Expression of *NbCYP71D20* and *NbPTI5* induced by VdEIX3 protein was greatly reduced in the *er-1* mutant. Data are shown as mean ± SD (Student’s *t*-test, ***P < 0.001).

To test whether additional PRR can recognize VdEIX3 besides NbEIX2 and NbRXEG1, we further generated *eix2 rxeg1* (*er*) double mutants by CRISPR/Cas9. We obtained two independent homozygous lines bearing premature termination of both genes. In both *er* lines, *eix2* contains a 1 bp insertion in the sgRNA region, while *rxeg1* contains deletions and insertions in both sgRNA regions (Figure 5A and Supplementary Figure 1). VdEIX3 was then expressed in *er* mutants by agroinfiltration. The *er* mutants showed no visible cell death 3 days post infiltration (Figure 5B). However, VdEIX3 eventually induced cell death 5 days post infiltration. This result indicates that the cell death-inducing activity of VdEIX3 was greatly impaired in *er* mutants.

To determine to what extent VdEIX3-induced resistance was impaired in *er* mutants, *P. capsici* was inoculated on *N. benthamiana* leaves pre-treated by buffer control or VdEIX3 protein. Similar to the cell death phenotype, pre-treatment of *er* mutants by VdEIX3 only slightly promoted plant resistance to *P. capsici* (Figure 5C). However, the average lesion areas of VdEIX3-treated regions in *er* mutants are less than that of buffer-treated regions, although they showed no significant difference (Figure 5C). In addition, the activation of PTI markers *NbCYP71D20* and *NbPTI5* was greatly reduced but not abolished in the *er-1* mutant (Figure 5D). These results suggest that NbEIX2 and NbRXEG1 are two major RLPs responsible for VdEIX3 detection and that additional PRR (s) with minor function is still present.

### The two divergent EIX-responding loci of *Nicotiana benthamiana* were acquired from different ancestors

**FIGURE 6:**
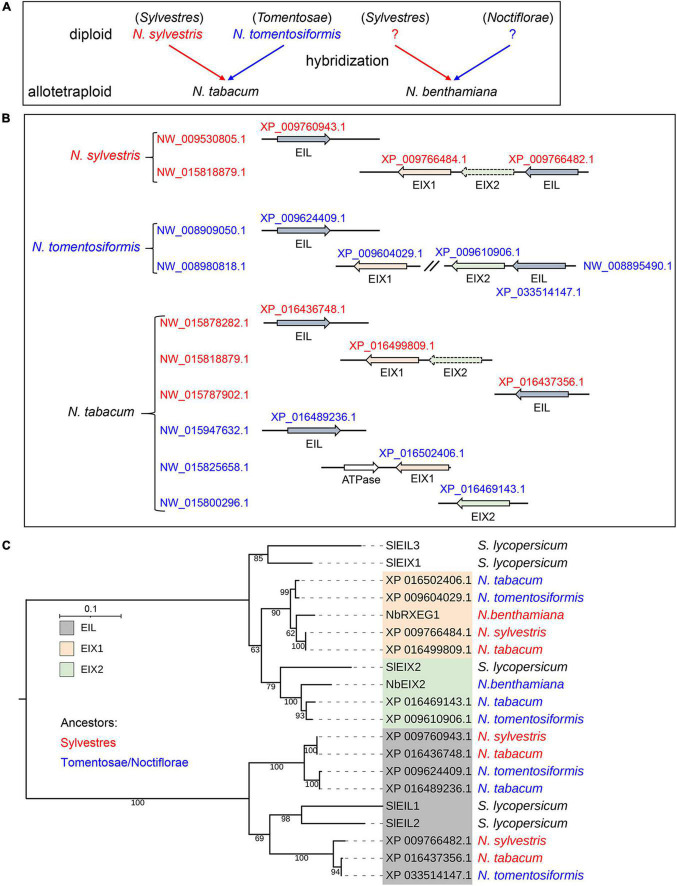
The two divergent EIX-responding loci of *Nicotiana benthamiana* were acquired from different ancestors. **(A)** The allotetraploid *N. tabacum* and *N. benthamiana* are hybrids of diploid *Nicotiana* species. Different progenitors are marked in blue and red, respectively. **(B)** NbEIX2 homologs identified in *N. sylvestris*, *N. tomentosiformis*, and *N. tabacum*. Accession numbers of the proteins and scaffolds shown in the figure are obtained from the GenBank database. The dotted arrow indicates pseudogene. **(C)** Phylogenetic analysis of NbEIX2 homologs from selected Solanaceae plants. NbEIX2 homologs are divided into three clades: EIX1, EIX2, and EIL. Sequences from different ancestors are marked in blue and red, respectively. The maximum likelihood phylogenetic tree was generated by W-IQ-TREE.

The current draft *N. benthamiana* genome assemblies are highly fragmented, and its completeness is about 96% (Bombarely et al., 2012; Bally et al., 2018), which probably results in the missing of gene information. The allotetraploid *N. tabacum* and *N. benthamiana* are hybrids of diploid *Nicotiana* species (Figure 6A). To trace the evolutionary trajectory of EIX-responding loci, we also identified NbEIX2 homologs in *N. tabacum* and its diploid progenitors *N. sylvestris* and *N. tomentosiformis*. Both diploid tobaccos contain and an EIX1 and an EIX2, among which EIX2 of *N. sylvestris* is a pseudogene (Figure 6B). The allotetraploid *N. tabacum* obtained the four EIX-responding loci which contain three NbEIX2 homologs totally (Figure 6B). In addition, both diploid tobaccos have two EILs, while *N. tabacum* has three or four EILs since the sequence of scaffold NW_015800296.1 is incomplete (Figure 6B).

To determine potential NbEIX2 homologs that are missing due to incomplete genome assemblies, we generated a phylogenetic tree of NbEIX2 homologs and EILs from these Solanaceae plants. This family of RLPs is divided into three clades: EIX1, EIX2, and EIL (Figure 6C). The phylogeny clearly showed that each clade contains EIX2 homologs from different ancestors. All of these Solanaceae plants have a single EIX2 member that recognizes xylanases (Figure 6C; Ron and Avni, 2004; Yin et al., 2021), while the number and function of EIX1 members are different. The diploid *S. lycopersicum* has one EIX1 member that functions as a decoy receptor by attenuating SlEIX2 signaling (Bar et al., 2010). The allotetraploid *N. tabacum* has two EIX1 members obtained from two progenitors, respectively (Figure 6B). *N. benthamiana* also contains two EIX1 members, however, one of which is from the *Noctiflora* progenitor bears premature termination (Figure 4A). The other EIX1 member is NbRXEG1, which regulates the perception of VdEIX3 (Figure 4) and PsXEG1 (Wang et al., 2018b). These results suggest that *N. benthamiana* obtained two divergent EIX-responding loci from different ancestors.

## Discussion

VdEIX3 is xylanase that belongs to glycosyl hydrolase family 11 (GH11) (Yin et al., 2021), and PsXEG1 is a GH12 xyloglucanase (Ma et al., 2015). These GH11 and GH12 enzymes are closely related and share similar protein structures (Törrönen et al., 1993), which likely explains our finding that NbRXEG1 can recognize both VdEIX3 and PsXEG1. An excellent example to support the phenomenon of NbRXEG1 is that the *Brassica napus* contains two variants of the same RLP, LepR3, and Rlm2, which confer resistance by recognizing AvrLm1 and AvrLm2, respectively, although the two avirulence effectors share very low similarity (Larkan et al., 2015). Previously, we showed that knockdown of *NbEIX2* by virus-induced gene silencing (VIGS) abolished VdEIX3-triggered immune responses (Yin et al., 2021). VIGS silences a gene by generating ∼21 nt small interfering RNAs from a ∼300 bp target sequence (Senthil-Kumar and Mysore, 2014), which possibly leads to off-target gene silencing due to the high similarity between NbEIX2 and NbRXEG1. The two types of MAMPs are widespread in microbes, including phytopathogenic bacteria, fungi, and oomycetes, which cause diverse diseases on important crops (Ma et al., 2015; Yin et al., 2021). In addition, many GH11 and GH12 proteins from important plant pathogens were reported to exhibit elicitor activity (Sella et al., 2013; Ma et al., 2015; Gui et al., 2017; Zhu et al., 2017; Frías et al., 2019; Yin et al., 2021). Thus, transfer of NbEIX2 or NbRXEG1 to crop plants will probably confer increased and broad-spectrum resistance. For instance, transgenic Solanaceae and rice plants showed broad-spectrum bacterial resistance by expressing the EFR receptor, which recognizes EF-Tu proteins from many bacterial pathogens (Lacombe et al., 2010). Importantly, NbEIX2 and NbRXEG1 function redundantly to detect GH11 and GH12 MAMPs.

Unlike animals which have the adaptive immune systems, plants rely on innate immunity to withstand various biotic attacks. To guarantee that plants detect extracellular dangerous signals effectively, several strategies are employed by PRRs to strengthen the defense. Strategy 1: two PRRs recognize different epitopes of a MAMP. For instance, two RLKs from the tomato, FLS2 and FLS3, recognize epitopes of bacterial flagellin termed flg22 and flgII-28, respectively (Chinchilla et al., 2006; Hind et al., 2016). Brassicaceae species use distinct PRRs to sense different immunogenic fragments from fungal endo-polygalacturonase (Zhang et al., 2021). Strategy 2: evolved PRRs perceive camouflaged MAMPs. The flg22 epitope of *Agrobacterium tumefaciens* evades FLS2 detection by most plants, while the FLS2 homolog from wild grape species *Vitis riparia* can detect the diverged flg22 (Furst et al., 2020). Likewise, soybean FLS2 perceives a polymorphic flg22 of *Ralstonia solanacearum*, flg22*^Rso^*, which avoids perception by other plants (Wei et al., 2020). Strategy 3: a family of PRRs redundantly recognize a ligand. For example, two *Arabidopsis* lectin receptor kinases P2K1 and P2K1 recognize extracellular ATP and contribute to innate immunity (Choi et al., 2014; Pham et al., 2020). The *Arabidopsis* tyrosine-sulfated peptide RGF7 is recognized by RLKs RGI4 and RGI5 and triggers innate immunity (Wang et al., 2021). In this study, two closely related LRR-RLPs NbEIX2 and NbRXEG1 redundantly detect fungal xylanase VdEIX3. *N. benthamiana* likely employs additional EIL member (s) to recognize VdEIX3 because *eix2 rxeg1* double mutants still can respond to VdEIX3 to a certain extent. Furthermore, the allotetraploid *N. benthamiana* likely has three or four EIL members according to the scenario of *N. tabacum*, which will be determined when a high-quality genome sequence is available.

In addition to redundant recognition of MAMPs by duplicated PRRs, functional divergence of PRRs from a family is another way to buffer attacks by various pathogens. *Arabidopsis* contains several LysM-containing PRRs that mediate innate immunity, among which LYK5 is the major receptor of fungal chitin (Cao et al., 2014), while LYM1 and LYM3 sense bacterial peptidoglycan (Willmann et al., 2011). Similarly, *Arabidopsis* has 17 *Catharanthus roseus* receptor-like kinase 1-like (CrRLK1L) receptors, several of which were shown to regulate plant immunity by recognizing distinct rapid alkalinization factor (RALF) peptides (Zhang et al., 2020). Here, we demonstrated that the functions of NbEIX2 and NbRXEG1 are redundant and diverged. NbEIX2, NbRXEG1, and potential EIL (s) can regulate VdEIX3-triggered immunity, while NbRXEG1 is required for the recognition of another type of MAMP PsXEG1 (Wang et al., 2018b). Furthermore, SlEIX1, the NbRXEG1 ortholog in tomato, plays a contrasting role in xylanase detection (Bar et al., 2010). The EIX family PRRs of *N. benthamiana* represent a distinct model for MAMPs recognition.

Rarely, a receptor senses different MAMPs to regulate immunity *via* distinct models. When NbRXEG1 recognizes PsXEG1, the co-receptor BAK1 is employed to activate immune responses (Wang et al., 2018b). However, VdEIX3 triggers immunity in a BAK1-independent manner (Yin et al., 2021). In *Arabidopsis*, the receptor-like kinase ERECTA recognizes the EPIDERMAL PATTERNING FACTOR (EPF) peptide EPF2 to regulate stomatal development, and ERECTA also recognizes EPF-like peptides EPFL4 and EPFL6 to regulate inflorescence development (Tameshige et al., 2016). The *Arabidopsis* CLAVATA3/ESR-RELATED (CLE) peptide CLE9/10 regulates stomatal and vascular development through HSL1 and BAM receptors, respectively (Qian et al., 2018). The HSL1 and BAM employ SOMATIC EMBRYOGENESIS RECEPTOR KINASES (SERKs) and CLV3 INSENSITIVE KINASEs (CIKs) as co-receptors, respectively (Qian et al., 2018; Hu et al., 2022). In contrast, when VdEIX3 was recognized by NbEIX2, BAK1 was released from the receptor complex (Yin et al., 2021), indicating the same signaling model. Likewise, both RGI4 and RGI5 require BAK1 to regulate RGF7 recognition (Wang et al., 2021). NbEIX2 and NbRXEG1 lack the cytoplasmic kinase present in RLKs, and the pivotal co-receptors BAK1 and SOBIR1 that are employed by most LRR-RLPs (Liebrand et al., 2014), are both dispensable for VdEIX3 recognition (Yin et al., 2021). Which RLK is the co-receptor of VdEIX3 remains unknown and is worth studying in the future. These findings reflect the specificity and complexity of innate immunity regulated by the *N. benthamiana* EIX family.

Polyploidization is a major force in the genome evolution of flowering plants (Van de Peer et al., 2020). Polyploidy results in duplicated genes that play important roles in crop domestication and disease resistance (Renny-Byfield and Wendel, 2014). A recent subgenome evolution analysis of allotetraploid angiosperms revealed that subgenomes of the allotetraploids exhibit unequal rates of protein evolution, showing separate evolutionary trajectories (Sharbrough et al., 2022). Consistently, *N. benthamiana* obtained two EIX-responding loci with diverged functions from different ancestors. Compared to the tomato EIX-responding locus that probably recognizes xylanases solely (Ma et al., 2015), the *N. benthamiana* EIX RLPs seem to undergo neofunctionalization and recognize two distinct MAMPs. Thus, the allotetraploid *N. benthamiana* exhibits somewhat defense heterosis at least in the perception of xylanase and PsXEG1. A similar scenario reported recently is that the allotetraploid *B. napus* contains four allelic wall-associated kinases (Rlm3, Rlm4, Rlm7, Rlm9) from progenitors *B. rapa* and *B. oleracea*, which confer race-specific resistance against blackleg disease by recognizing variable avirulence effectors (Larkan et al., 2020; Haddadi et al., 2022). Besides immune receptors, other duplicated proteins by hybridization also confer improved resistance. For instance, WRKY transcription factor homologs from the artificial allotetraploid *Arabidopsis suecica* show different regulatory networks of defense genes, which contributes to increased defense in allotetraploid (Abeysinghe et al., 2019). However, duplicated genes or improper activation of immune receptors by hybridization might lead to hybridization incompatibility, which is also known as hybrid necrosis (Li and Weigel, 2021). Interestingly, we noticed that *N. benthamiana* obtained four EIX RLPs but only two of them are functional since the others bear premature termination. Potentially, *N. benthamiana* avoids hybrid necrosis by discarding two EIXs.

In summary, our results demonstrate that the LRR-RLP NbRXEG1, which was previously shown to recognize PsXEG1 and related MAMPs (Wang et al., 2018b), also participates in the detection of xylanases. We also showed that the allotetraploid *N. benthamiana* obtained divergent EIX-responding LRR-RLPs from different ancestors. Therefore, a family of LRR-RLPs with divergent functions redundantly recognizes two types of MAPMs that are widespread in diverse pathogens (Supplementary Figure 2), which confers robust and broad-spectrum pattern-triggered immunity. Co-transfer of NbEIX2 and NbRXEG1 to crop plants is a promising strategy for resistance breeding.

## Data availability statement

The datasets presented in this study can be found in online repositories. The names of the repository/repositories and accession number(s) can be found in the article/Supplementary material.

## Author contributions

LW and DD conceived and designed the experiments. NW generated the *Nicotiana benthamiana* mutants using CRISPR/Cas9. NW, ZY, YZ, and ZL performed all other experiments and analyzed data. ZY performed the bioinformatics analyses and wrote the manuscript. All authors contributed to the article and approved the submitted version.

## Footnotes

1
https://www.genome.jp/tools-bin/clustalw
