# Supplementary material for: Two divergent immune receptors of the allopolyploid Nicotiana benthamiana reinforce the recognition of a fungal microbe-associated molecular pattern VdEIX3
Source: Front Plant Sci. 2022 Aug 15;13:968562. doi: 10.3389/fpls.2022.968562 (PMC9421165; doi:10.3389/fpls.2022.968562)
Supplement: Supplementary file 2 [file Data_Sheet_1.docx]

**Supplementary information**


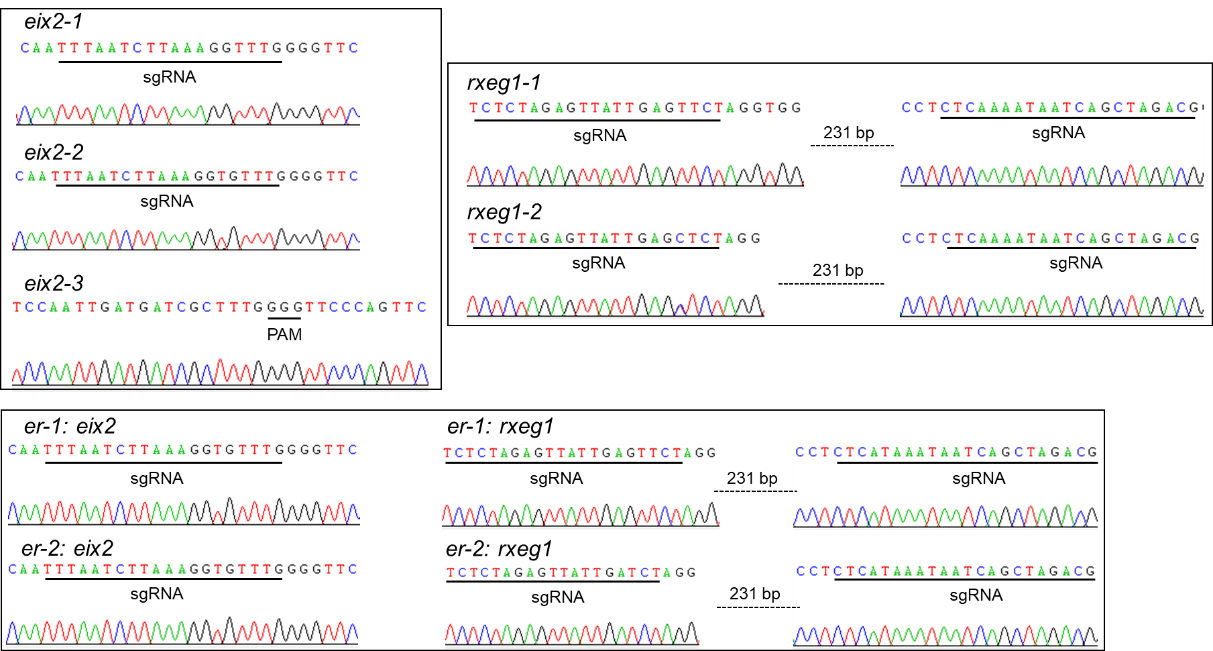


Supplementary Figure 1. Sanger sequencing chromatograms of CRISPR mutants in this study.


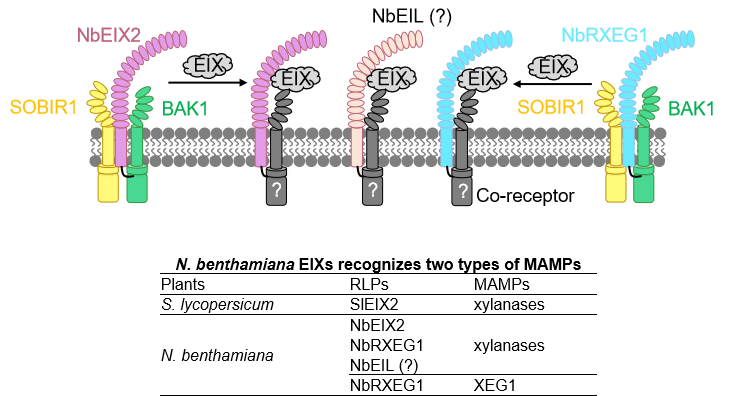


Supplementary Figure 2. Proposed model showing NbEIX2 and NbRXEG1 synergistically recognize xylanases (EIX).

Supplementary File 1. The deleted region in the *eix2-3* mutant.

*The 60 bp region deleted in the *eix2-3* mutant is marked in red.

>NbEIX2

ATGGATAAAAGGAAATATCCAAGATTAGATCATTTCCTTGTTACTTGGTCTTTATTGCTCTTACAGACAGCTTTAGGATTAACTTCAAGAGAAGTTAACAAAACCATGTGTATACAAAAGGAGAGAGATGCTCTTCTTGAGTTCAAAAGAGGCCTTATTGATGATTTTGATCGATTATCAACATGGGGCGATGAAGAAGATAAAAAAGAATGCTGCAAATGGAAGGGTATTGAATGTGACAAAAGAAGTGGTCATGTAACTGTTCTTGATCTTCACACTGAGGTTTCATGTCCAGTCCGTTCTTGTTTTGCGCCAATGTTGACAGGTAAACTTAGTCCTTCTCTACTTGAGTTGCACCATCTGAATTACTTGGACCTCAGTCATAATGGATTTGACAAAATTGAAATACCAAGATTCATATCCTCTCTTAAGAGACTTGAGTATCTGAACCTCTCATCTTCAGATTTTTCTGGTGTAATTCCTACACAGTTAAAGAATCTAACTTCTTTGAGGATTCTTGATCTTGGGAACAATAATCAGCTAATAGTAAAGGACCTTGGGTGGCTTTCTTATCTCTCCTCGCTAGAGATCTTGCGTCTAGGTGGTAACGACTTCCAAGCAAGCAATTGGTTCCAAGAGATAACAAAGGTACCTTCACTGAAAGAACTCGACTTGAGTGTTTGTGGTCTCTCTAAATTTGTTCCATCTCCAGCTGATTTAGTCAAGTCTTCTTTGATCTCTCTTTCTGTTCTTCATTTATGTTGTAATCAGTTTACTTCTTCAGCTGAATATAGCTGGTTATTCAATTTCAGCACAAGCTTAACTAGCATAGACCTCTCCAATAATCAGCTGGACGGTCCAATTGATGATCGCTTTGGGAGCTTGATGTATCTTGAGCATCTTAAACTTGCTGATCAATTTAATCTTAAAGGTGTTGGGGTTCCCAGTTCTTTTGGGAACTTGACACGTTTACGTTATCTGGACATATCTAGCACTCGGACATACCAATGGCTTCCTGAGTTGTTTCTCAGGTTATCAGGAAGCAGGAAAACACTTGAGGTTTTGGGGTTGAACGACAACTCAATGTTTGGTTCATTGGTTAACGTCACAAGATTTTCAGCTTTAAAGAGATTATACCTGCAGAACAATGTGCTGAATGGTTTTTTCATGGAAAGATTTGGACAAGTTTCGAGTCTCGAGTATCTAGACTTGTCTGATAACCAAATGAGAGGGTCATTACCAGATTTAGCATTGTTTCCATCAATGAGAGAGTTGCATCTAGGTTCTAATCAATTTCAAGGGAGGATACCACAAGGTATTGGAAAACTTTCACAGCTTAGAATTTTGGACGTCTCGTCCAATAGGCTGGAAGGATTACCAGAAAGTATGGGGCAACTATCGAACCTGGAAAGTTTTGATGCCTCTTACAATGCCCTGAAGGGTACAATCACTGAGTCCCATCTTTCAAACCTCTCCAGTTTAGTGTATTTGGACTTATCGTTCAACTCGTTGGCTTTGAAGACGAGCTTTGATTGGCTTCCTCCTTTTCAGCTTCAATTTATAAACCTTCCATCTTGCAATTTGGGACCTTCTTTCCCCAAGTGGCTTCAAAGTCAAAACAACTATACTGTTCTTGATATCTCTCTTGCAAATCTATCAGATGCGCTACCAAGTTGGTTCTCTGATCTTCCTCTCAATTTAAAGATTCTGAATCTCTCTAACAACCATATCAGCGGAAGAGTTTCTGAGTTTATAGTGAATAAACAAGACTACATGGTTATAGATTTAAGTTCTAACAACAATTCAGGACCTTTGCCGCTAGTTCCTATCAATGTCCGAATATTTTACCTACATAAAAATAAGTTTTCCGGATCCACTTCTTCCATTTGTAAAAGTACAACAGGAGGTGCCACTTCCGTTGACTTGTCACACAACCAATTTTCAGGAGAACTTCCTGATTGTTGGATGAATATGAGTAATCTAGTTGTTCTTAATCTAGCCTATAACAATTTCTCTGGAAAACTTCCACAGTCATTAGGTTCCTTGGAAAGTTTGGAGGCATTATACATACGCCAGAACAGTTTTAACGGGATGTTGCCTTCTTTCTCACAATGTCAATCATTGCAAATCTTGGATCTTGGAGGGAATAAGTTGACAGGAAGAATCCCAGCATGGATAGGTAATGATCTACTCAACTTGCGTATTCTAAGCCTACGGTTCAACAAATTCTATGGTAGCATTCCATCGATCATTTGTCAGCTTCAATTTCTTCAGATACTGGACATTTCAGCAAATGGATTATCCGGGAAAATTCCACAATGCTTCAACAATTTTACTTTATTGCATCAAGAAAATGGTTCTGGTGAGTCGATGGAATTTTTAGTCCAGCTTGACTATTTGCCTCGTTCATACTTGTACATAGGCAATTTATTGGTTCAATGGAAAAACCAGGAGGCTGAGTACAAGAATCCTTTATTATATCTGAAGGCTATTGATCTTTCAAGTAATAAATTGGTCGGAAATATTCCTAAAGAGATAGCTGAAATGAGAGGATTGAAATCTTTGAACCTTTCAAGAAATGATCTGAATGGAAGTATCATTGAAGGAATCGGTCAAATGAAGATGTTGGAGTCACTTGACCTGTCAAGAAACCAGCTTTCTGGTATGATTCCTAAAGGCCTTGCTAACTTGACTTTTATTGGTGTTTTGGACTTGTCAAACAACCACTTATCAGGGAGAATTCCATCAAGCACTCAACTCCAAACTTTTGAGACATCATCCTATAGTGGTAATGCTCAACTCTGCGGCCCTCCTCTTGAAGAATGTCCTGGATTTGCTCCTCCTAGCCCCCGTATCAATCATGGTAGCAATATCAATCCCCAAGAACTTGGTGATGATGATGAGTTTCCGTCTCTGGAGTTTTATATATCAATGGTGCTCGGTTTCTTCGTTGCATTTTGGGGAATCTTGGGCTGTTTAATTGTCAACCGTGCTTGGAGGAATGCCTACTTCACATTCTTAATGGACACGAAGAATTGGCTCGCTATGATATCAAGAGTCTGCTTTACAAGACTGAAGGGAAAGCTAAGGGCCTCATAA

>NbEIX2

MDKRKYPRLDHFLVTWSLLLLQTALGLTSREVNKTMCIQKERDALLEFKRGLIDDFDRLSTWGDEEDKKECCKWKGIECDKRSGHVTVLDLHTEVSCPVRSCFAPMLTGKLSPSLLELHHLNYLDLSHNGFDKIEIPRFISSLKRLEYLNLSSSDFSGVIPTQLKNLTSLRILDLGNNNQLIVKDLGWLSYLSSLEILRLGGNDFQASNWFQEITKVPSLKELDLSVCGLSKFVPSPADLVKSSLISLSVLHLCCNQFTSSAEYSWLFNFSTSLTSIDLSNNQLDGPIDDRFGSLMYLEHLKLADQFNLKGVGVPSSFGNLTRLRYLDISSTRTYQWLPELFLRLSGSRKTLEVLGLNDNSMFGSLVNVTRFSALKRLYLQNNVLNGFFMERFGQVSSLEYLDLSDNQMRGSLPDLALFPSMRELHLGSNQFQGRIPQGIGKLSQLRILDVSSNRLEGLPESMGQLSNLESFDASYNALKGTITESHLSNLSSLVYLDLSFNSLALKTSFDWLPPFQLQFINLPSCNLGPSFPKWLQSQNNYTVLDISLANLSDALPSWFSDLPLNLKILNLSNNHISGRVSEFIVNKQDYMVIDLSSNNNSGPLPLVPINVRIFYLHKNKFSGSTSSICKSTTGGATSVDLSHNQFSGELPDCWMNMSNLVVLNLAYNNFSGKLPQSLGSLESLEALYIRQNSFNGMLPSFSQCQSLQILDLGGNKLTGRIPAWIGNDLLNLRILSLRFNKFYGSIPSIICQLQFLQILDISANGLSGKIPQCFNNFTLLHQENGSGESMEFLVQLDYLPRSYLYIGNLLVQWKNQEAEYKNPLLYLKAIDLSSNKLVGNIPKEIAEMRGLKSLNLSRNDLNGSIIEGIGQMKMLESLDLSRNQLSGMIPKGLANLTFIGVLDLSNNHLSGRIPSSTQLQTFETSSYSGNAQLCGPPLEECPGFAPPSPRINHGSNINPQELGDDDEFPSLEFYISMVLGFFVAFWGILGCLIVNRAWRNAYFTFLMDTKNWLAMISRVCFTRLKGKLRAS*
